# Effectiveness of a Remote Intervention Program for Self-Management Behaviors in Adolescents and Young Adults With Inflammatory Bowel Disease Based on the Self-Determination Theory: Randomized Controlled Trial Across 2 Centers

**DOI:** 10.2196/79370

**Published:** 2025-12-05

**Authors:** Yangfan Zhu, Yueyue Chen, Jinjiu Hu, Xin Wan, Hong Guo, Xiaoqin Zhou, Delin Wang, Xin Zhang, Xianlan Zheng, Hao Wang

**Affiliations:** 1 Department of Gastroenterology Chongqing General Hospital Chongqing China; 2 Mental Health Education and Counseling Center Chongqing Institute of Foreign Studies Chongqing China; 3 Department of Nursing Children’s Hospital of Chongqing Medical University Chongqing, Yuzhong District China; 4 Department of Gastroenterology Children’s Hospital of Chongqing Medical University Chongqing, Yuzhong District China; 5 Department of Big Data Center for Children’s Medical Care Children’s Hospital of Chongqing Medical University Chongqing, Yuzhong District China

**Keywords:** adolescent, young adult, inflammatory bowel disease, self-management, social support, anxiety, depression

## Abstract

**Background:**

The prevalence of inflammatory bowel disease (IBD) has been on the rise, with adolescents and young adults experiencing the highest incidence rates. For these young patients, self-management behaviors are critical to maintaining disease remission and improving quality of life and yet their current self-management status remains suboptimal.

**Objective:**

This study aimed to evaluate the impact of a remote and multicomponent intervention program (integrating health education, solution-focused intervention, peer support, and mindfulness training) on the self-management behaviors and related mental health outcomes in young adults and adolescents with IBD.

**Methods:**

From July 2024 to January 2025, we conducted a 2-arm, single-blind randomized controlled trial. Adolescents and young adults with IBD (aged 13-24 years) were recruited from gastroenterology wards of 2 tertiary hospitals in Chongqing, China, and randomized 1:1 to the intervention (extra multicomponent program) or control (routine care) group. Outcomes (self-management behaviors, perceived social support, basic psychological needs, anxiety, depression, and disease activity) were assessed postintervention (T1) and 12 weeks later (T2). Intention-to-treat analysis was used: normally distributed data via mixed-design analysis of variance, and nonnormal data via rank sum and Friedman tests.

**Results:**

A total of 74 participants (mean age 18.95, SD 2.96 years; 53/74, 72% males) were enrolled (37 per group) with no baseline differences. Compared with the control group, the intervention group showed significantly higher self-management scores at both T1 (mean difference –16.676, 95% CI –24.370 to –8.982; *P*<.001, η^2^=0.206) and T2 (mean difference –14.189, 95% CI –22.594 to –5.784; *P*=.001, η^2^=0.136), along with higher perceived social support scores at T1 (mean difference –9.000, 95% CI –13.932 to –4.068; *P*=.001, η^2^=0.155) and T2 (mean difference –6.649, 95% CI –11.890 to –1.407; *P*=.01, η^2^=0.082). Basic psychological needs scores were also higher in the intervention group at T1 (mean difference –4.946, 95% CI –8.323 to –1.569; *P*=.005, η^2^=0.106) and T2 (mean difference –3.946, 95% CI –7.720 to –0.172; *P*=.04, η^2^=0.057), while anxiety (T1: *P*=.04; T2: *P*=.007) and depression (T1: *P*=.048; T2: *P*=.03) scores were lower. In addition, the intervention group had a higher disease activity remission rate at T2 (*P*=.03).

**Conclusions:**

This study extended the application of self-determination theory to adolescents and young adults with IBD, offering a novel theoretical basis for self-management practice in this population. It was shown that this multicomponent intervention was a valuable addition to standard care in enhancing self-management behaviors and related mental health outcomes while lowering disease activity. In addition, its alignment of intrinsic behavioral motivation with nurse-driven clinical settings boosted clinical operability of the intervention.

**Trial Registration:**

Chinese Clinical Trial Registry ChiCTR2400086703; https://www.chictr.org.cn/showproj.html?proj=235313

## Introduction

### Background

Inflammatory bowel disease (IBD) is a chronic, immune-mediated inflammatory condition of the gastrointestinal tract, comprising ulcerative colitis, Crohn disease, and unclassified types [1]. The global prevalence of IBD has been rising [2], which results in notable economic and health care burdens [3]. With enhanced diagnostic capabilities and rapid urbanization, the incidence of IBD has significantly risen in China, making it the country with the highest prevalence in Asia [4]. By 2025, the number of individuals affected by IBD could reach 1.5 million in China [5]. The highest incidence of IBD is among adolescents and young adults [6].

Currently, IBD is a lifelong condition with no definitive cure. Manifestations such as repeated diarrhea, fecal blood, stomach ache, and severe tiredness greatly affect the life quality of adolescents and young adults [7], with self-management behaviors being crucial in enhancing life quality and disease prognosis [8]. Self-management behaviors encompass patient actions aimed at sustaining and enhancing their health via various self-guided actions, covering areas such as medical, emotional, and role management [9].

The adolescent and young adult phase represents a critical transition from childhood to adulthood, characterized by substantial physiological and social role transformations [10]. Young individuals with IBD encounter dual challenges: managing their medical condition while adapting to social role changes [10]. Consequently, researchers have highlighted that self-management behaviors of adolescents and young adults with IBD need to be improved [11-13]. Therefore, effective interventions are urgently needed to enhance self-management behaviors in this population.

However, existing interventions for adolescents and young adults with IBD often concentrate on isolated aspects of self-management and demonstrate considerable heterogeneity in results [14]. Moreover, these interventions [14], which are typically led by psychologists, do not align with China’s clinical practice. In China, clinical nurses primarily assume responsibility for patient self-management. Consequently, it is critically important to develop an all-encompassing and effective program for self-management behaviors of adolescents and young adults with IBD, particularly within a nurse-led clinical environment.

The formation and sustainability of self-management behaviors are underpinned by underlying motivational mechanisms. The self-determination theory [15] serves as a pivotal framework in behavioral studies, playing a crucial role in predictive model construction and intervention design [16]. This theory highlights that fulfilling basic psychological needs (competence, autonomy, and relatedness) is indispensable for fostering motivation and sustaining behaviors [15].

Based on the self-determination theory, our research team conducted a preliminary study [17] on the influencing factors of self-management behaviors in adolescents and young adults with IBD. The study [17] revealed that perceived social support would influence self-management behaviors through the mediating effects of basic psychological needs and emotional issues, indicating that enhancing perceived social support, satisfying basic psychological needs, and alleviating emotional issues were crucial for improving self-management behaviors. To identify effective strategies for these improvements, we conducted a systematic review of evidence [18] in self-management interventions for this population. The review found that multicomponent interventions were the most effective approach. Health education was necessary to increase knowledge and satisfy the need for competence; peer support could significantly enhance perceived social support and satisfy the need for relatedness; group-based mindfulness training could effectively relieve emotional problems; and remote interventions were shown to improve adherence to intervention among adolescents and young adults. In addition, solution-focused intervention [19], which complements self-determination theory by addressing the basic psychological needs [20], has been commonly applied in nursing in the form of short-term groups to enhance self-management behaviors among adolescents and young adults [21].

Building on our preliminary study [17] and systematic review [18], we designed a multicomponent intervention program tailored to enhance self-management behaviors in adolescents and young adults with IBD. This program was delivered through short-term remote group sessions and integrated health education, solution-focused intervention, peer support, and mindfulness training to address the basic psychological needs underlying self-management behaviors, thereby promoting the initiation and maintenance of self-management behaviors.

### Objectives

This research primarily aimed to evaluate the effectiveness of this intervention program over standard care in fostering self-management behaviors among adolescents and young adults with IBD. The ancillary goals included assessing its effectiveness in improving the perceived social support and basic psychological needs, diminishing levels of anxiety and depression, and lessening disease activity in this group.

## Methods

This study was implemented in accordance with the predefined trial protocol [22] and was reported according to the CONSORT (Consolidated Standards of Reporting Trials) reporting guidelines [23].

### Study Design and Setting

Conducted between July 2024 and January 2025, this research entailed a double-center, single-blind, 2-arm randomized controlled trial in gastroenterology units of 2 tertiary hospitals (1 pediatric hospital and 1 general hospital) in Chongqing, China. Chongqing stands as a municipality under direct administration and a central national city in China.

### Participants

Inclusion criteria were diagnosis of ulcerative colitis or Crohn disease [24], age ranging from 13 to 24 years [25], and ability to provide informed consent and express oneself clearly. Exclusion criteria were having severe intellectual impairment; pregnancy; history of cancer or active cancer diagnosis; currently receiving psychiatric medications, therapy, or other psychological intervention; and refusal to participate. Withdrawal criteria were voluntary withdrawal for personal reasons, accompanied by an exit interview to elucidate the reasons for withdrawal; and loss of contact.

### Informed Consent and Baseline Assessment

A researcher (DLW) recruited participants from inpatients at the gastroenterology wards of the 2 hospitals in July 2024. Eligible patients were identified by reviewing daily admission lists and approached directly in their wards. The researcher provided a verbal explanation of the study and obtained written informed consent from participants or guardians. For participants younger than 18 years, parental consent was required before obtaining the adolescents’ consent. The recruiting researcher was not involved in the delivery of the intervention.

The baseline assessment was administered via a unique web link to the questionnaire hosted on the Wenjuanxing platform (a Chinese online survey tool compliant with data privacy regulations) within 24-48 hours after obtaining informed consent. Following completion of research ethics and survey administration training, the researchers (JJH and XW) conducted a baseline assessment, which included (1) collection of general information, including age, gender, residence, ethnicity, annual household income, current educational background, main caregiver, disease type and duration, and surgical history; and (2) assessment of outcome variables, as described in “Outcome Assessment” section.

### Sample Size

The sample size was calculated using PASS software (version 16.0; NCSS Limited Liability Company), with parameters derived from a related previous study [26]. Specifically, that study [26] reported a mean difference of 10.1 in self-management behavior scores between the 2 comparison groups, with a corresponding pooled SD of 5.54. Setting a significance level (α) of .05 (2-tailed) and a desired statistical power of 0.8 (80%), the initial calculated sample size was 47 participants. After accounting for a projected dropout rate of 19% (9/47), the final minimum sample size was determined to be 56 participants, with no fewer than 28 individuals in each group (intervention group and control group).

### Randomization and Blinding

Following enrollment, participants were assigned sequential numbers. A researcher independent of the study team then randomly allocated them to the control and intervention groups at a 1:1 ratio, using random numbers generated by the RAND function in Excel software (Microsoft Corp). To ensure allocation concealment, allocation results were stored in sequentially numbered, sealed envelopes maintained by an independent research assistant external to the research team and opened only at the time of intervention initiation. Participants, the recruiter, outcome evaluators, and the data analyst were blinded to group assignments.

### Intervention

#### Control Group

Routine care was provided to the control group, including face-to-face health education during hospitalization and at discharge, a telephone follow-up within 1 week of discharge, and real-time doctor-patient communication via WeChat (a widely used social media app in China). While not formally standardized across all settings, this communication method aligns with local clinical practices in our region for maintaining postdischarge engagement.

#### Intervention Group

The intervention group received the remote intervention program in addition to routine care. To develop this program, a stakeholder workshop was organized. For this workshop, 2 adolescents and young adults with IBD (aged 17 years with a 3-year disease history and 21 years with a 5-year disease history, respectively) and 13 health providers (see Table S1 in Multimedia Appendix 1 for details) were invited to discuss and revise the draft program, culminating in the finalization of a multicomponent remote group intervention program. In addition, data on the health care providers’ judgment bases and their familiarity were collected (Tables S2 and S3 in Multimedia Appendix 1). Based on these judgment bases and familiarity levels, we further calculated the authority coefficient of the health care providers’ judgments. The specific calculation principles and results are provided in the “Health Care Providers’ Judgments” section of Multimedia Appendix 1.

The intervention program is detailed in Table S4 in Multimedia Appendix 1. This program consisted of 9 weekly sessions facilitated through a remote conferencing platform (Tencent Meetings software; Tencent Holdings Limited). This 9-week duration aligned with the semester vacation of Chinese students, which was expected to increase their participation enthusiasm. With the exception of the initial and final weeks, which focused on starting and ending the program, every weekly session comprised these components:

1. Health education: This component covered medication, dietary management, physical exercise, disease monitoring, vaccination, and home care procedures. Its objective was to enhance self-management knowledge and satisfy the need for competence.
2. Solution-focused intervention: This involved goal-setting discussions, exception-seeking questions, scaling questions, miracle questions, and relationship-oriented questions, aiming to comprehensively boost the satisfaction of basic psychological needs.
3. Peer support: Participants engaged in discussions and shared their experiences and insights, fulfilling the need for relatedness. Volunteers from local patient organizations were also invited to share their stories, encouraging and motivating participants to open up.
4. Mindfulness training: This component aimed to relax the emotion and enhance the perception of internal and external resources.

#### Regulating Quality

To guarantee the effectiveness of the program’s execution within the intervention group, these steps were implemented:

#### Preparation of Intervention Materials

To aid participants in fully grasping the program, a uniform manual, a tailored canvas bag, and a pen (illustrated in Figure S1 in Multimedia Appendix 1) were created and disseminated.

#### Implementation of the Intervention

The nurse (YFZ) received training from the psychological counselor (YYC). The counselor participated throughout the intervention process to provide quality supervision and guidance.

The intervention adopted a group discussion approach. Using the online conferencing Tencent Meetings software, participants were randomly divided into smaller groups of 2-3 members. After the group discussions, a collective sharing session was held to enhance engagement.

#### Reinforcement of Intervention Effects

Relevant homework assignments were assigned to reinforce and solidify the effects of the intervention.

After each intervention activity, adolescents were required to complete a feedback scale to rate their satisfaction on a scale of 1-5.

For participants unable to attend sessions in real time, the intervention was documented via video recording of the full group session. Researcher YFZ supervised these participants to ensure that they viewed the recorded videos within 1 week of the session.

### Outcome Assessment

Information was gathered by researchers (JJH and XW) through the self-reporting questionnaire on the Wenjuanxing platform, with the exception of disease activity, which underwent external evaluation via the electronic medical record system and phone interviews.

#### Measurement of the Main Outcome Indicator: Self-Management Behavior

We used the Self-Management Behavior Scale of Inflammatory Bowel Disease, developed by Chinese scholars [27], to assess the self-management behaviors of the participants. The scale encompasses 7 dimensions: medication management, dietary practices, disease monitoring, emotional regulation, physical exercise, daily life, and resource utilization, and it comprises 36 items. Responses are gauged on a 5-point scale, ranging from 1 (never) to 5 (always). The Cronbach a coefficient of the scale was 0.945 in the original study and 0.941 in this study. Chinese scholars commonly use this scale to evaluate self-management in patients with IBD [28].

#### Measurement of Secondary Outcome Indicators

##### Basic Psychological Needs

This study used the Chinese version of the Basic Psychological Needs Satisfaction Scale [29], adapted from the original one [30]. This version contains 9 items and 3 dimensions, namely, autonomy, competence, and relatedness. The rating for each item ranges from 1 (strongly disagree) to 7 (strongly agree). The Cronbach a coefficient of this scale was 0.86 in the original study and 0.941 in this study. This version of this scale has been widely used [31].

##### Perceived Social Support

This study used the Chinese version of the Perceived Social Support Scale [32], adapted from the original one [33]. The scale consists of 12 items divided into 3 dimensions: family support, friend support, and other support, and is assessed on a 7-point scale from 1 (strongly disagree) to 7 (strongly agree). The Cronbach a coefficient of the scale was 0.88 in the original study and 0.943 in this study. This scale has been widely used [34].

##### Anxiety

The Generalized Anxiety Disorder 7-item Scale (GAD-7) was used to assess the severity of anxiety over the past 2 weeks. Comprising 7 elements, this item is evaluated on a scale ranging from 0 (not at all) to 3 (almost daily). The total score of GAD-7 ranges from 0 to 21, with score ranges interpreted as follows: 0-4 points indicate no significant anxiety symptoms, 5-9 points denote mild anxiety symptoms, and a score of ≥10 points indicates the generalized anxiety symptoms [35]. The Chinese version of the GAD-7 has been widely used in clinical practice [35]. The Cronbach a coefficient of this scale was 0.937 in this study.

##### Depression

The Patient Health Questionnaire-9 (PHQ-9) was used to assess the level of depression in the past 2 weeks. It contains 9 items, which are scored on a scale from 0 (not at all) to 3 (almost every day). The total score of the PHQ-9 ranges from 0 to 27, with established interpretive criteria: 0-4 points indicate no significant depressive symptoms, 5-9 points denote mild depressive symptoms, and a score of ≥10 points is indicative of moderate to severe depression symptoms [36]. The Chinese version of the PHQ-9 is a reliable measure of depressive symptoms in clinical practice [36]. The Cronbach a coefficient of this scale was 0.920 in this study.

##### Disease Activity Level

For participants with Crohn disease, the Pediatric Crohn’s Disease Activity Index was applied to those younger than 18 years, while the Crohn’s Disease Activity Index was used for those aged 18 years and older. For participants with ulcerative colitis, the Pediatric Ulcerative Colitis Activity Index was used for those younger than 18 years, and the Simple Clinical Colitis Activity Index was used for those aged 18 years and older. Using these measurements, the severity of disease activity was categorized into remission, mild, moderate, or severe [37].

### Evaluation Schedule

Outcome indicators of participants were assessed at baseline (T0), immediately after the intervention (T1), and 12 weeks after the intervention (T2). For validity, an interim analysis was conducted at T1: no significant differences in primary or secondary outcomes would have resulted in a decision to stop T2 follow-up; if differences existed, results remained confidential until all data collection was complete (in line with the blinded protocol), with details available in the study protocol [22].

### Statistical Analysis

Statistical analyses were conducted using SPSS software (version 26.0; IBM Corp). To examine categorical data across 2 groups, either the chi-square test or the Fisher exact test was used, with findings displayed in terms of frequencies and percentages. Normality tests were performed on continuous variables to determine the suitable statistical techniques. Information showing a normal distribution underwent analysis via the *t* test and was presented as mean (SD). In contrast, data that did not follow a normal distribution were evaluated using the rank sum test and presented as median (IQR).

For normally distributed data assessed at multiple time points within a group, mixed-design analysis of variance was used. Effect sizes were presented as partial eta-squared (η^2^ ). The value of η^2^ ranges from 0 to 1 and can be interpreted as small (η^2^ ≥0.01), medium (η^2^≥0.06), and large effects (η^2^≥0.14) [38]; for non–normally distributed data, the Friedman test was applied, with Bonferroni correction used for post hoc multiple comparisons. The significance level (α) was set at .05. For Bonferroni correction, the adjusted significance threshold was calculated as 0.05 divided by the number of comparisons (n=3), resulting in a corrected statistical significance level of *P*<.017. Subgroup analyses were not conducted due to the small sample size in this study.

A Little’s Missing Completely At Random test was performed to evaluate the missing mechanism (*χ*^2^_14_=10.82; *P*=.63), confirming that the data were missing completely at random. Missing data were addressed through multiple imputation methods. The analysis of this study adhered to the principles of intention-to-treat analysis.

### Ethical Considerations

Approval for the research was granted by the ethics review boards of the Children’s Hospital of Chongqing Medical University and Chongqing General Hospital (approval numbers: file nos. 2023,395 and KYS2024-008-01). No ethical exemption was applied. Written informed consent was obtained from all participants (with guardians providing consent for those younger than 18 years), and the informed consent forms are available in Multimedia Appendix 1. No secondary analysis was planned, with ethics approval for no extra consent. Data were deidentified (unique codes) and stored encrypted. No participant compensation was provided. No identifiable images were included; future use requires consent and form uploads.

### Result

#### Overview

**Figure 1:**
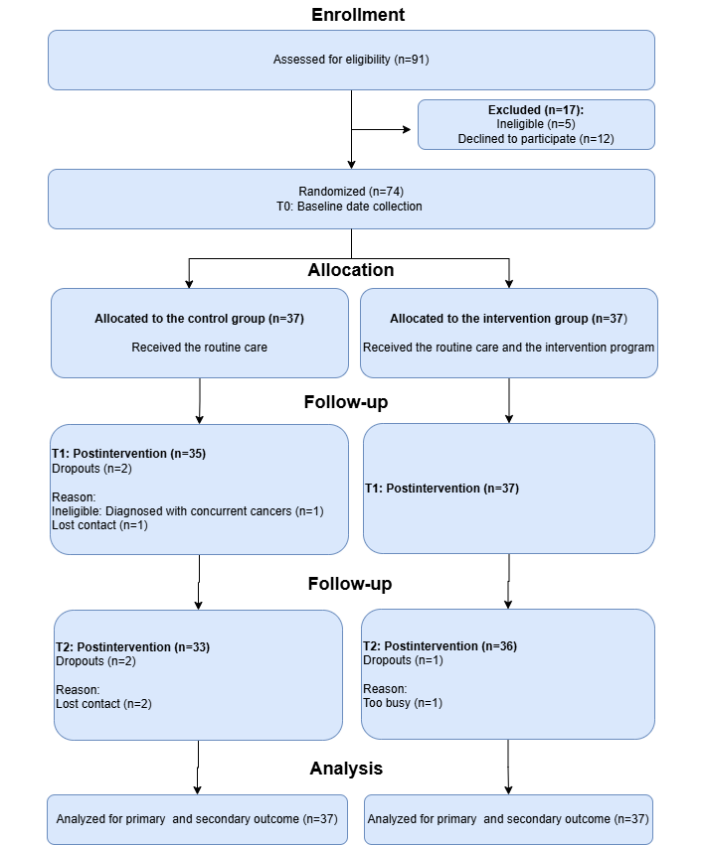
Flow diagram: a randomized controlled trial for self-management behaviors in adolescents and young adults with inflammatory bowel disease, Chongqing, China (July 2024 to January 2025).

Initially, 91 potential participants were identified, with 17 excluded: 2 ineligible for failing to meet the inclusion age, 3 ineligible due to unconfirmed diagnosis, and 12 declining participation for personal reasons. As a result, 74 participants were recruited, with 37 assigned to the intervention group and 37 to the control group. Of the participants, 74 (100%) completed the evaluation at T0; 72 (97.3%) completed the evaluation at T1, with 2 cases of missing data (2.7% missing rate); and 69 (93.2%) completed the evaluation at T2, resulting in 5 cases of missing data (6.8% missing rate). A flow diagram of the study is shown in Figure 1. No important harms or unintended effects were observed in either group.

In the intervention group, the mean real-time participation rate during the 9 online intervention sessions was 79.52% (95% CI 66.8%-92.2%), while the recorded video-viewing rate was 20.48% (95% CI 8.1%-32.9%). The satisfaction score was mean 4.97 (SD 0.08, 95% CI 4.94-5.00) on a 5-point scale.

#### Baseline Characteristics

The age of the participants was mean 18.95 (SD 2.96) years. Males constituted 71.62% (53/74) of the sample. Table 1 displays the sociodemographic details and clinical traits of the participants, revealing no significant difference between the intervention and control groups at baseline.

**Table 1:** Sociodemographic information and clinical characteristics of participants in a randomized controlled trial for self-management behaviors in adolescents and young adults with inflammatory bowel disease, Chongqing, China (July 2024 to January 2025).

Participant characteristics		All (N=74)	Control group (N=37)	Intervention group (N=37)	Chi-square (*df*)/*t* test (*df*)		*P* value	
Age (years), mean (SD)		18.95 (2.96)	19.41 (2.88)	18.49 (3.00)	1.345 (72)^a^		.18	
**Disease type, n (%)**						1.138 (1)^b^		.48
	Ulcerative colitis	65 (87.84)	31 (83.78)	34 (91.89)				
	Crohn disease	9 (12.16)	6 (16.22)	3 (8.11)				
**Sex, n (%)**						0.066 (1)^b^		.80
	Male	53 (71.62)	27 (72.97)	26 (70.27)				
	Female	21 (28.38)	10 (27.03)	11 (29.73)				
**Ethnicity, n (%)**						0.725 (1)^b^		.67
	Han	68 (91.89)	35 (94.59)	33 (89.19)				
	Minority	6 (8.11)	2 (5.41)	4 (10.81)				
**Residence, n (%)**						0.398 (1)^b^		.53
	Urban	62 (83.78)	30 (81.08)	32 (86.49)				
	Rural	12 (16.22)	7 (18.92)	5 (13.51)				
**Annual household income (CNY^c^: yuan; 1 USD^d^= 7.08 CNY), n (%)**						3.939 (2)^b^		.15
	≤50,000	44 (59.46)	20 (54.05)	24 (64.86)				
	50,001-150,000	25 (33.78)	16 (43.24)	9 (24.32)				
	150,000	5 (6.76)	1 (2.70)	4 (10.81)				
**Current educational background, n (%)**						2.286 (3)^b^		.54
	Middle school	10 (13.51)	4 (10.81)	6 (16.22)				
	High school	28 (37.84)	12 (32.43)	16 (43.24)				
	College	27 (36.49)	15 (40.54)	12 (32.43)				
	Postcollege	9 (12.16)	6 (16.22)	3 (8.11)				
**Main caregiver, n (%)**						4.32 (2)^b^		.12
	Parents	50 (67.57)	22 (59.46)	28 (75.68)				
	Grandparents	10 (13.51)	8 (21.62)	2 (5.41)				
	Self	14 (18.92)	7 (18.92)	7 (18.92)				
**Disease duration (years), n (%)**						0.057 (1)^b^		.81
	≤2	29 (39.19)	15 (40.54)	14 (37.84)				
	>2	45 (60.81)	22 (59.46)	23 (62.16)				
**Have undergone IBD^e^-related surgery, n (%)**						0.259 (1)^b^		.61
	Yes	22 (29.73)	10 (27.03)	12 (32.43)				
	No	52 (70.27)	27 (72.97)	25 (67.57)				
**Type of hospital attended, n (%)**						0.093 (1)^b^		.76
	Pediatric	13 (17.57)	6 (16.22)	7 (18.92)				
	General	61 (82.43)	31 (83.78)	30 (81.08)				

^a^*t* test.

^b^ Chi-square.

^c^CNY: Chinese Yuan.

^d^USD: United States dollar.

^e^IBD: inflammatory bowel disease.

#### Effects of the Intervention on the Primary Outcome

**Figure 2:**
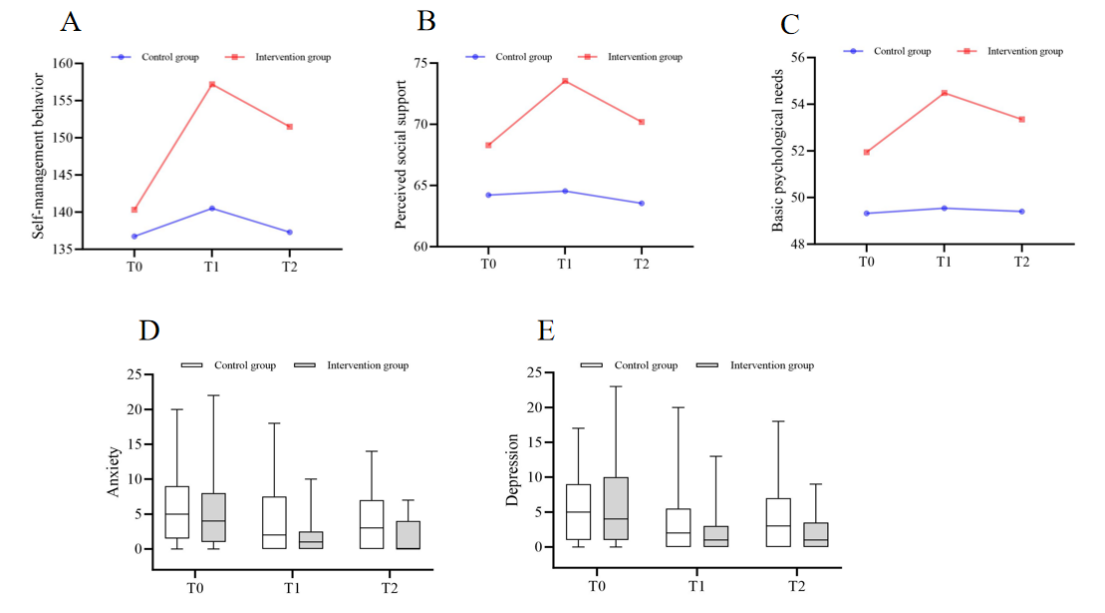
Between-group differences in changes of all study variables (A: self-management behaviors; B: perceived social support; C: basic psychological needs; D: anxiety; and E: depression) at different time points in a randomized controlled trial for adolescents and young adults with inflammatory bowel disease, Chongqing, China (July 2024 to January 2025): control group (n=37) versus intervention group (n=37).

As shown in Table 2, regarding self-management behaviors, a significant time × group interaction was observed (*F*_interaction effect_=8.339; *P*<.001); between-group comparisons showed no difference at T0 (95% CI –12.728 to 5.539; *P*=.435, η^2^=0.008) but significant superiority of the intervention group at T1 (95% CI –24.370 to –8.982; *P*<.001, η^2^=0.206) and T2 (95% CI –22.594 to –5.784; *P*=.001, η^2^=0.136); and within-group analyses revealed no changes in the control group (*P*=.16, η^2^=0.050) but significant differences in the intervention group (*P*<.001, η^2^=0.426). For detailed within-group comparisons across different time points, see Table S5 in Multimedia Appendix 1. The trend of these results is illustrated in Figure 2A. For the analysis of the scores across various dimensions of self-management behaviors, refer to Table S6 in Multimedia Appendix 1.

**Table 2:** Between-group and within-group differences in self-management behaviors, perceived social support, and basic psychological needs in a randomized controlled trial for adolescents and young adults with inflammatory bowel disease, Chongqing, China (July 2024 to January 2025) at T0, T1, and T2.

Indicators						T0		T1		T2		*F* test (*df*)		*P* value		η^2^
**Self-management behaviors^a^**																
				Control group (n=37), mean (SD)	136.73 (19.65)		140.51 (17.25)		137.30 (21.48)		1.853 (2)		.16		0.050	
				Intervention group (n=37), mean (SD)	140.32 (19.76)		157.19 (15.93)^b^		151.49 (14.01)^b,c^		26.354 (2)		<.001		0.426	
				Mean difference (SE)	–3.595 (4.582)		–16.676^d^ (3.859)		–14.189^d^ (4.216)		N/A^e^		N/A		N/A	
				95% CI	–12.728 to 5.539		–24.370 to –8.982		–22.594 to –5.784		N/A		N/A		N/A	
				*F* test (*df*)	0.616 (1)		18.667 (1)		11.325 (1)		N/A		N/A		N/A	
				*P* value	.44		<.001		.001		N/A		N/A		N/A	
				η^2^	0.008		0.206		0.136		N/A		N/A		N/A	
**Perceived social support^f^**																
				Control group (n=37), mean (SD)	64.22 (10.49)		64.54 (11.81)		3.54 (12.30)		0.276 (2)		.76		0.008	
				Intervention group (n=37), mean (SD)	68.30 (10.86)		73.54 (9.33)^b^		70.19 (10.22)^c^		8.351 (2)		.001		0.190	
				Mean difference (SE)	–4.081 (2.483)		–9.000^d^ (2.474)		–6.649^d^ (2.629)		N/A		N/A		N/A	
				95% CI	–9.030 to 0.868		–13.932 to –4.068		–11.890 to –1.407		N/A		N/A		N/A	
			*F* test (*df*)		2.702 (1)		13.231 (1)		6.394 (1)		N/A		N/A		N/A	
		*P* value			.11		.001		.01		N/A		N/A		N/A	
	η^2^				0.036		0.155		0.082		N/A		N/A		N/A	
**Basic psychological needs^g^**																
	Control group (n=37), mean (SD)				49.32 (7.58)		49.54 (7.93)		49.41 (8.81)		0.025 (2)		.98		0.001	
	Intervention group (n=37), mean (SD)				51.95 (7.74)		54.49 (6.59)^b^		53.35 (7.41)		3.115 (2)		.049		0.081	
	Mean difference (SE)				–2.622 (1.782)		–4.946^d^ (1.694)		–3.946^d^ (1.893)		N/A		N/A		N/A	
	95% CI				–6.173 to 0.930		–8.323 to –1.569		–7.720 to –0.172		N/A		N/A		N/A	
	*F* test (*df*)				2.165 (1)		8.524 (1)		4.345 (1)		N/A		N/A		N/A	
	*P* value				.15		.005		.04		N/A		N/A		N/A	
	η^2^				0.029		0.106		0.057		N/A		N/A		N/A	

^a^*F*_group effect_=9.404, *P*=.003; *F*_time effect_=18.534, *P*<.001; and *F*_interaction effect_=8.339, *P*<.001.

^b^Statistically significant difference compared with T0 within group with Bonferroni correction (*P*<.017).

^c^Statistically significant difference compared with T1 within group with Bonferroni correction (*P*<.017).

^d^*P*<.05.

^e^N/A: not applicable.

^f^*F*_group effect_=8.880, *P*=.004; *F*_time effect_=5.363, *P*=.007; and *F*_interaction effect_=3.264, *P*=.04.

^g^*F*_group effect_=5.956, *P*=.02; *F*_time effect_=1.724, *P*=.18; and *F*_interaction effect_=1.231, *P*=.30.

#### Effects of the Intervention on Secondary Outcomes

##### Effects of the Intervention on Perceived Social Support

As shown in Table 2, regarding perceived social support, a significant time × group interaction was observed (*F*_interaction effect_=3.264; *P*=.04); between-group comparisons showed no difference at T0 (95% Cl –9.030 to 0.868; *P*=.105, η^2^=0.036) but significant superiority of the intervention group at T1 (95% CI –13.932 to –4.068; *P*=.001, η^2^=0.155) and T2 (95% CI –11.890 to –1.407; *P*=.014, η^2^=0.082); and within-group analyses revealed no changes in the control group (*P*=.76, η^2^=0.008) but significant differences in the intervention group (*P*=.001, η^2^=0.190). For detailed within-group comparisons across different time points, see Table S5 in Multimedia Appendix 1. The trend of these results is illustrated in Figure 2B. For the analysis of the scores across various dimensions of perceived social support, refer to Table S7 in Multimedia Appendix 1.

##### Effects of the Intervention on Basic Psychological Needs

As shown in Table 2, regarding basic psychological needs, no significant time × group interaction was observed (*F*_interaction effect_=1.231; *P*=.30); between-group comparisons showed no difference at T0 (95% CI –6.173 to 0.930; *P*=.146, η^2^=0.029) but significant superiority of the intervention group at T1 (95% CI –8.323 to –1.569; *P*=.005, η^2^=0.106) and T2 (95% CI –7.720 to –0.172; *P*=.04, η^2^=0.057); and within-group analyses revealed no changes in the control group (*P*=.98, η^2^=0.001) but significant differences in the intervention group (*P*=.049, η^2^=0.081). For detailed within-group comparisons across different time points, see Table S5 in Multimedia Appendix 1. The trend of these results is illustrated in Figure 2C. For the analysis of the scores across various dimensions of basic psychological needs, refer to Table S8 in Multimedia Appendix 1.

##### Effects of the Intervention on Anxiety

The Mann-Whitney *U* test was conducted to compare anxiety scores between the 2 groups at different time points, with the results summarized in Table 3. At T0, there was no significant difference detected among the groups (*P*=.75, *z*=–0.321). At T1 and T2, the intervention group demonstrated statistically lower scores than the control group (*P*=.04, *z*=–2.096; *P*=.007, *z*=–2.69). Within-group comparisons revealed that the control group’s anxiety scores exhibited a statistically significant overall difference (*P*=.007, *χ*^2^_2_=9.894), with post hoc analysis indicating that the score at T2 was significantly lower than that at T0 (*P*<.017). For the intervention group, anxiety scores also showed a statistically significant overall difference (*P*<.001, *χ*^2^_2_=32.463), with post hoc analysis demonstrating that scores at both T1 and T2 were significantly lower than that at T0 (*P*<.017). The trend of these results is illustrated in Figure 2D.

##### Effects of the Intervention on Depression

The analysis of depression scores is shown in Table 3. At T0, the 2 groups showed no significant difference (*P*=.92, *z*=–0.098). At T1 and T2, the intervention group demonstrated statistically lower scores than the control group (*P*=.048, *z*=–1.981; *P*=.03, *z*=–2.115). Within-group comparisons revealed that the control group’s depression scores exhibited a statistically significant overall difference (*P*=.03, *χ*^2^_2_=6.764). However, the post hoc analysis showed no statistically significant differences between time points in the control group (*P*>.017). For the intervention group, depression scores also showed a statistically significant overall difference (*P*<.001, *χ*^2^_2_=15.228), with post hoc analysis demonstrating that scores assessed at T1 and T2 were markedly less than that at T0 (*P*<.017). The trend of these results is illustrated in Figure 2E.

**Table 3:** Between-group and within-group differences in anxiety and depression scores in a randomized controlled trial for adolescents and young adults with inflammatory bowel disease, Chongqing, China (July 2024 to January 2025) at T0, T1, and T2.

Indicators		T0	T1	T2	Chi-square (*df*)	*P* value
**Anxiety**						
	Control group (n=37), median (IQR)	5.00 (2.00-9.00)	2.00 (0.00-7.00)	3.00 (0.00-7.00)^a^	9.894 (2)	.007
	Intervention group (n=37), median (IQR)	4.00 (1.00-8.00)	1.00 (0.00-2.00)^a^	0.00 (0.00-4.00)^a^	32.463 (2)	<.001
	*z*	–0.321	–2.096	–2.69	N/A^b^	N/A
	*P* value	.75	.04	.007	N/A	N/A
**Depression**						
	Control group (n=37), median (IQR)	5.00 (1.00-9.00)	2.00 (0.00-5.00)	3.00 (0.00-6.00)	6.764 (2)	.03
	Intervention group (n=37), median (IQR)	4.00 (1.00-9.00)	1.00 (0.00-3.00)^a^	1.00 (0.00-3.00)^a^	15.228 (2)	<.001
	*z*	–0.098	–1.981	–2.115	N/A	N/A
	*P* value	.92	.048	.04	N/A	N/A

^a^Statistically significant difference compared with T0 within group with Bonferroni correction (*P*<.017).

^b^N/A: not applicable.

##### Effects of the Intervention on Disease Activity

Disease activity between the 2 groups was evaluated using the Mann-Whitney *U* test, as detailed in Table 4. Findings showed negligible variance in disease activity between the groups at T0 and T1 (*P*=.44, *z*=–0.769; *P*=.16, *z*=–1.403). At T2, a higher percentage of participants in the intervention group experienced remission than those in the control group, showing statistically significant differences (*P*=.03, *z*=–2.231).

**Table 4:** Comparison of disease activity between groups in a randomized controlled trial of adolescents and young adults with inflammatory bowel disease, Chongqing, China (July 2024 to January 2025) at T0, T1, and T2.

		All (n=74)	Control group (n=37)	Intervention group (n=37)	*z*		*P* value	
**T0, n (%)**						–0.769		.44
	Remission	57 (77.03)	26 (70.27)	31 (83.78)				
	Mild activity	10 (13.51)	6 (16.22)	4 (10.81)				
	Moderate activity	4 (5.41)	2 (5.41)	2 (5.41)				
	Severe activity	3 (4.05)	3 (8.11)	0 (0.00)				
**T1** **, n (%)**						–1.403		.16
	Remission	64 (86.49)	30 (89.19)	34 (91.89)				
	Mild activity	8 (10.81)	5 (13.51)	3 (8.11)				
	Moderate activity	1 (1.35)	1 (2.70)	0 (0.00)				
	Severe activity	1 (1.35)	1 (2.70)	0 (0.00)				
**T2** **, n (%)**						–2.231		.03
	Remission	66 (89.19)	30 (81.08)	36 (97.30)				
	Mild activity	8 (10.81)	7 (18.92)	1 (2.70)				

## Discussion

### Principal Findings

Self-determination theory has been widely validated for improving self-management behaviors in other populations with chronic diseases [16,39]. A key innovation of this study was its first application of this theory to adolescents and young adults with IBD, offering a novel theoretical framework for clinical interventions targeting this population. Based on the mechanisms underlying the formation and sustainability of self-management behaviors [17], this study developed a remote multicomponent program, integrating health education, solution-focused intervention, peer support, and mindfulness training This intervention program showed significant effects in enhancing self-management behaviors, strengthening perceived social support, and fulfilling basic psychological needs among adolescents and young adults with IBD, while also mitigating their anxiety, depression, and disease activity. Notably, unlike traditional in-person intervention, this remote program could offer greater flexibility. The favorable real-time participation rate and satisfactory feedback score in the intervention group indicated that the program was well received by participants.

Regarding self-management behaviors, the intervention group demonstrated superiority over routine care, highlighting that the intervention program should serve as a valuable and beneficial complement to routine care. Routine care primarily relies on one-way health education. As a complex, multidimensional construct (encompassing disease, emotional, and role management), self-management behaviors cannot be fully improved by routine care’s typical one-way health education [40]. Critically, most self-management intervention studies have been led by specialized psychotherapists [14], rendering them unsuitable for nurse-led clinical settings. Although this study used a multidisciplinary and multicomponent intervention, its overall nurse-led approach could enhance clinical feasibility and offer insights for regions with similar clinical contexts.

In perceived social support, the intervention group exhibited a significant advantage over the control group. This advantage in the intervention group could be plausibly attributed to the intervention’s multicomponent design. Unlike extant literature [41] that predominantly used peer support to modulate psychological outcomes in patients with IBD, this study innovatively integrated peer support with solution-focused intervention, transcending passive reciprocal assistance to proactively cultivate participants’ capacity to identify, mobilize, and optimize inherent support resources within their lived contexts.

In addition, this study revealed that the scores of competence and relatedness (2 dimensions of basic psychological needs) in the intervention group were higher than those in the control group at T1 and T2 (see Table S8 in Multimedia Appendix 1). However, the autonomy dimension did not achieve significance either within groups or between groups at all time points, as elaborated in Table S8 in Multimedia Appendix 1. Although theoretical literature [20] posited that solution-focused intervention could enhance the satisfaction of basic psychological needs, its practical application should be contextualized within specific cultural backgrounds. Within an Asian cultural context, parental authority and overprotection often hinder the development of adolescents’ autonomy [42]. Against this cultural backdrop, the autonomy of participants in this study proved challenging to foster in the absence of parental involvement. From the perspective of self-determination theory, this study framed autonomy around attaining self-independence. Notably, the program might not account for adolescents’ potential to view “relying on parents” as an autonomous choice. Future research should thus reframe objectives to explore how adolescents use parental support to meet autonomy needs, rather than solely emphasizing self-independence.

Although the intervention program outperformed routine care in reducing anxiety and depression in the participants, within-group analyses showed that the control group also had significantly lower anxiety scores at T2 than at T0. This finding implied that routine care had a certain positive impact on emotion. Alternatively, it could be inferred that the potential for self-growth among adolescents and young adults with IBD was consistent with other research [43] that reported posttraumatic growth trends in this population. This observation corroborated the use of a solution-focused approach, which guided participants to recognize intrinsic resources (inherently present in participants, with the intervention facilitating awareness of personal strengths). Furthermore, this study advanced posttraumatic growth theory from phenomenological description to intervention-based empirical validation in this population, providing an entry point for investigating the mediating mechanisms of the disease-related stress and self-growth pathway.

Finally, there were no significant changes in disease activity levels at T1; however, a significant improvement was observed at T2, providing empirical support for the influence of mental health on disease activity, consistent with “gut-brain axis” theory [44]. This result suggested that the psychological intervention did not yield immediate disease benefits and sustained engagement was needed to modulate brain-gut cross talk, clinically guiding health care providers and patients to set realistic expectations for long-term adherence. Notably, while other study [45] has also reported that adding psychological interventions to routine care effectively alleviates disease activity, these interventions were predominantly led by specialized psychologists. In contrast, the nurse-led model of this study could render gut-brain axis-informed care accessible in regions with limited access to psychologists.

### Limitations

However, this study still has some limitations. The long-term effectiveness of this study remains to be further verified, as follow-up was limited to 12 weeks. It is recommended to conduct long-term follow-up to determine whether the intervention effect is sustainable in the long run. Furthermore, the sample in this study mainly consisted of individuals with Crohn disease (65/74, 87.84%), males (53/74, 71.62%), and urban populations (62/74, 83.78%). Although this is in line with the epidemiological characteristics of IBD in China [5], the imbalance limits generalizability. Efficacy in subgroups such as rural residents or patients with ulcerative colitis remains untested, as these groups may face unique barriers (eg, limited access to remote resources in rural areas) affecting outcomes. In addition, while the sample size calculation confirmed sufficient statistical power for the primary outcomes, the modest sample size may hinder detection of small but clinically meaningful effects (eg, the autonomy dimension of basic psychological needs).

### Conclusions

Based on the self-determination theory, this study developed a short-term, group-based, remote, and multicomponent intervention program, integrating health education, peer support, solution-focused intervention, and mindfulness training. The program demonstrated improvements in self-management behaviors, perceived social support, and basic psychological needs among adolescents and young adults with IBD, while also alleviating their anxiety, depression, and disease activity. Theoretically, this study validated the application of a combination of multiple intervention components under the guidance of self-determination theory in adolescents and young adults with IBD. Practically, it was shown that the nurse-led remote intervention was feasible and accessible. Future research should verify the program’s long-term effectiveness and expand to more balanced samples to enhance generalizability; optimizing the intervention to address unmet autonomy needs could further boost its clinical use.

## Data Availability

The data supporting the findings of this study can be obtained upon reasonable request from the corresponding author. Note that the data are not publicly accessible due to privacy and ethical considerations.

## Appendix

Multimedia Appendix 1 Details of expert authority evaluation for the intervention, the full multicomponent intervention program, pairwise comparisons of outcome measures across time points, and detailed statistical results of self-management behaviors, perceived social support, and basic psychological needs (by specific dimensions) between the intervention and control groups, along with an informed consent form and a supplementary figure.

Multimedia Appendix 2 CONSORT-eHEALTH checklist (V 1.6.1).

## Abbreviations

CONSORT: Consolidated Standards of Reporting Trials
GAD-7: Generalized Anxiety Disorder 7-item Scale
IBD: inflammatory bowel disease
PHQ-9: Patient Health Questionnaire-9
