# Supplementary material for: Effectiveness of a Remote Intervention Program for Self-Management Behaviors in Adolescents and Young Adults With Inflammatory Bowel Disease Based on the Self-Determination Theory: Randomized Controlled Trial Across 2 Centers
Source: J Med Internet Res. 2025 Dec 5;27:e79370. doi: 10.2196/79370 (PMC12717511; doi:10.2196/79370)
Supplement: Multimedia Appendix 1 [file jmir_v27i1e79370_app1.docx]

**Health care providers’ judgments involved in the stakeholder workshop:**

The judgment bases encompassed four dimensions: practical experience, theoretical analysis, reference to domestic and international literature, and subjective perception. For each dimension, experts rated the degree of influence as “High,” “Medium,” or “Low” with corresponding assigned values: practical experience (0.5, 0.4, 0.3), theoretical analysis (0.3, 0.2, 0.1), reference to domestic and international literature (0.1, 0.1, 0.1), and subjective perception (0.1, 0.1, 0.1).

Familiarity with the intervention program was categorized into five levels: very familiar, relatively familiar, moderately familiar, slightly familiar, and unfamiliar, with assigned values of 0.9, 0.7, 0.5, 0.3, and 0.1, respectively. The authority coefficient was calculated using the formula: authority coefficient = (score of judgment basis + score of familiarity) / 2. This coefficient ranges from 0 to 1, with higher values indicating a higher level of expert authority.

The final analysis showed a mean score of 0.954 for judgment bases and 0.769 for familiarity; the overall authority coefficient was 0.862, demonstrating a high level of expert authority. Detailed results of the providers' judgment bases and familiarity levels are presented in **Table S2** and **Table S3**.

**Table S1.** Characteristics of health care providers (n = 13) involved in the stakeholder workshop**.**

| **Number** | **Professional field** | **Institution type** |
| --- | --- | --- |
| 5 | Nursing care in IBD | Pediatric hospitals (2); Adult hospitals (3) |
| 5 | Diagnosis and treatment in IBD | Pediatric hospitals (2); Adult hospitals (3) |
| 1 | Pediatric chronic disease management | Pediatric hospitals |
| 1 | Evidence-based medicine | Adult hospitals |
| 1 | Psychology | Pediatric hospitals |

**Table S2.** Results of judgment basis.

| **Main judgment basis** | **High (Frequency, n)** | **Medium (Frequency, n)** | **Low (Frequency, n)** |
| --- | --- | --- | --- |
| Practical experience | 9 | 4 | 0 |
| Theoretical analysis | 11 | 2 | 0 |
| Reference to literature | 9 | 4 | 0 |
| Subjective perception | 0 | 7 | 6 |

**Table S3.** Results of judgment familiarity**.**

| **Familiarity level** | **Very familiar (Frequency, n)** | **Relatively familiar (Frequency, n)** | **Moderately familiar (Frequency, n)** | **Unfamiliar (Frequency, n)** |
| --- | --- | --- | --- | --- |
| Frequency | 2 | 10 | 1 | 0 |

**Table S4.** The multicomponent intervention program in self-management behaviors for adolescents and young adults with IBD.

| No. | Theme | Content |
| --- | --- | --- |
| Session #1 | Initial meeting | 1. Introduction of the group theme: An overview of the group contract, clarifying activity norms and confidentiality principles, as well as explaining the specific process of the intervention activity. 2. Health education: Overview of IBD and self-management behaviors. 3. Self-introduction of group members: Each member begins with "I am" and writes several sentences about themselves, followed by a brief sharing of their disease journey and current life situation. 4. Viewing a patient story video and engage in a collective reading of a mindfulness poem. |
| Session #2 | Insights into the Solution-Focused Approach (SFA） | 1. Health education of medication management. 2. SFA activity: An explanation of the development background and steps of the Solution-Focused Approach. One person will be guided as an example to experience the steps of the SFBT, addressing potential issues in medication self-management. Participants were randomly pair up to discuss their current concerns based on the steps, followed by collective sharing session. 3. Mindful breathing training. 4. Homework: Record the completion status of medication adherence goals over the course of the week, noting any issues related to medication management, including missed doses, incorrect dosing, or delayed treatment. And outline subsequent solutions. |
| Session #3 | Viewing Adversity from a Different Perspective | 1. Homework sharing and positive feedback 2. Health education of dietary management. 3. SFA activities. ①“*Adversity Story*”: Facilitated a session where adolescents help protagonists in a story identify resources within their challenges. ②“*My Lifeline 1.0*”: Participants are encouraged to create a timeline on a blank paper, plotting their age (0~100 years) on the horizontal axis and their life satisfaction (0~100 points) on the vertical axis. They identified the intersection point of their current age and life satisfaction, share their current challenges and goals, and recall three past experiences of adversity. For each setback, they noted intersection point of the age it occurred and the corresponding life satisfaction score. Connecting all these points formed “*My Lifeline 1.0*”. Participants discussed in pairs or small groups of 2~3, followed by a collective sharing session. 4. Mindfulness senses training. 5. Homework: Keep a food diary for one week, identifying current issues and subsequent solutions |
| Session #4 | Exploring Exceptions | 1. Homework sharing and positive feedback. 2. Exercise health education. 3. SFA activity- “*My Life Line 2.0*”: Participants were asked to create a life coordinate axis, identifying the intersection point of their current age and life satisfaction. They observed changes in life satisfaction compared to the previous session and discussed the reasons behind these changes. Participant were asked to recall three successful experiences in their lives, marking intersection point of the age at which each success occurred and the corresponding life satisfaction levels. All intersection points were connected, creating a visual representation of “*My Life Line 2.0*”. Participants discussed in pairs or small groups of 2~3, followed by a collective sharing session. 4. Mindfulness Standing Training. 5. Homework：Document a weekly exercise diary, identifying current issues and subsequent solutions. |
| Session #5 | Setting quantifiable goals | 1. Homework sharing and positive feedback. 2. Health education of symptom monitoring and growth assessments. 3. SFA activity- the Fable “*The Three Alarms*”; Facilitated discussions among adolescents to share the small steps they have taken to address current challenges. Participants discussed in pairs or small groups of 2~3, followed by a collective sharing session. 4. Mindfulness *STOP* training: ①Stop: Pause to acknowledge any negative emotions you may be experiencing. ②Take a Breath: Focus your attention on the present moment through mindful breathing. ③ Observe: Notice what is happening around you and reflect on your current emotions. ④Proceed: Calmly move forward with your actions. 5. Homework: Record disease symptoms and growth development indicators. |
| Session #6 | Imagining the Miracles | 1. Homework sharing and positive feedback. 2. Health education of vaccination management 3. SFA activity – “*Messages Across Time*”: This activity guides adolescents to imagine a future day when the issues have been resolved and to reflect on the different feelings and changes they would experience at that time. Participants are encouraged to write a message to their present selves, conveying these future experiences and insights. Participants discussed in pairs or small groups of 2~3, followed by a collective sharing session. 4. Mindful non-discriminative awareness training. 5. Homework: Record the vaccination status of each vaccine and assess the suitable vaccines for boosting immunity and. |
| Session #7 | Emotion Decoding | 1. Homework sharing and positive feedback. 2. Health education on practical home self-management practices for enteral feeding, stoma care, perianal care, and subcutaneous injection of biologics. 3. SFA activity: ① Share the impacts of IBD on daily life. ② Watch the video “*Understanding Our Emotions: Seven Key Insights*” (https://www.bilibili.com/video/BV13p4y167d4/); and engage in a connecting activity: link different emotions with their underlying needs. ③ Given a case scenario, discuss what we truly want to express when uncomfortable emotions arise, such as anger, sadness, or frustration, and how we can meet the needs behind these emotions. Participants discussed in pairs or small groups of 2~3, followed by a collective sharing session. 4. Mindful breathing training. 5. Document your experiences with self-care practices at home (if any). Additionally, record one of the most unpleasant events that occurred within the past week, including physical sensations and your emotional feelings or thoughts during the event, and the methods you employed to regulate your emotions. |
| Session #8 | Role Understanding | 1. Homework sharing and positive feedback. 2. Social Role Health Education: Conducting personality assessments and explain methods for self-awareness. 3. SFA activity. ① “*I Am 2.0*”: Start each sentence with “I am” and write 10 sentences related to relationships. ② “My Relationship Circle”: Add connecting lines between different “circles” to represent intimacy and conflict, mapping out internal and external support systems (family, friends, society). ③ “*Coping Strategy Map*”: Create a map to represent current challenges, past successful experiences, internal and external resources available now and in the future, and small steps that can be taken to achieve goals. 4. Mindful breathing training. 5. Homework: Document the most pleasant event that occurred within the week, including the participants involved and their relationships, as well as the physical sensations, internal feelings, or thoughts experienced during the event. |
| Session #9 | Farewell and Reflection | 1. Homework sharing and positive feedback. 2. Summary and sharing activity: Reviewing the process of activity, sharing participants’ feelings and gains from participation, and concluding with farewell and expressions of gratitude and well-wishes. |

Note: Health education was designed to address the needs for competence; peer discussion and support aimed at fulfilling the needs for relatedness; and the SFA activity was structured to meet the needs for competence, relatedness, and autonomy simultaneously.


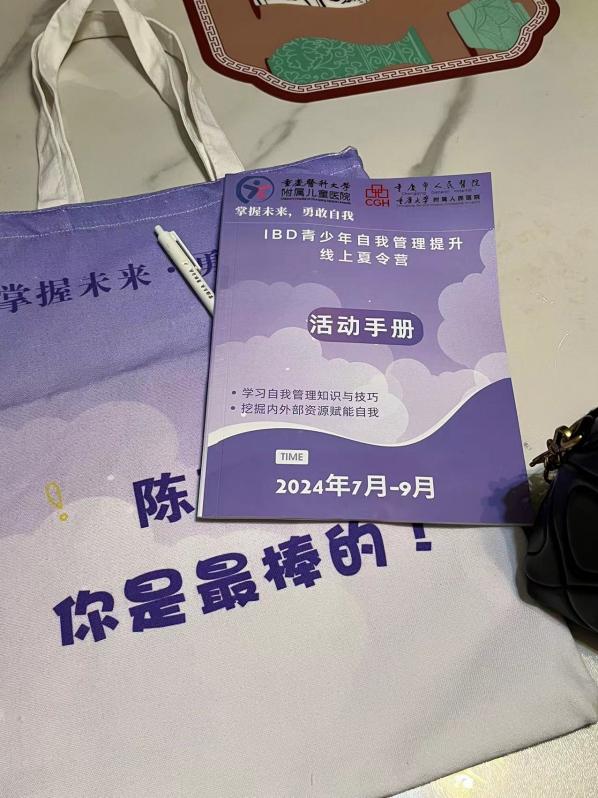


**Figure S1.** Standardized manual, customized canvas bag, and pen.

#### Informed Consent

The principal investigator or relevant research personnel have verbally informed me of the information related to this research, and I have also read the above - mentioned written information.

I have been given ample opportunity to discuss the above - mentioned research, and the questions I raised have received clear and definite answers.

I agree to participate in this research and understand that my participation is entirely voluntary.

I understand that I can withdraw from the research at any time, and my withdrawal will not affect my future medical treatment.

I am aware that I can contact the researchers at any time when encountering research - related issues, and I can contact the Office of the Medical Research Ethics Committee of this hospital when encountering issues related to my own rights and interests, and I have been provided with accurate contact information.

For the purpose of voluntarily signing this informed consent form, I agree that my personal information data, including my medical information data, will be used in the manner described above.

I know that I will receive a copy of this informed consent form.

Patient’s signature: ____________
Date: ____ / ____ / ____
Contact number: ____________

**Table S5.** Pairwise comparison of time points per control and intervention group.

| **Indicators** | **Group** | **Time point** | | ***MD*** | ***SE*** | **95% CI** | |
| --- | --- | --- | --- | --- | --- | --- | --- |
|  |  |  |  |  |  | **Upper limit** | **Lower limit** |
| Self-Management Behaviors | Control group | T0 | T1 | -3.784 | 2.313 | -9.452 | 1.885 |
|  |  |  | T2 | -.568 | 2.686 | -7.151 | 6.016 |
|  |  | T1 | T2 | 3.216 | 2.190 | -2.153 | 8.585 |
|  | Intervention group | T0 | T1 | -16.865^*^ | 2.313 | -22.533 | -11.196 |
|  |  |  | T2 | -11.162^*^ | 2.686 | -17.746 | -4.579 |
|  |  | T1 | T2 | 5.703^*^ | 2.190 | 0.333 | 11.072 |
| Perceived social support | Control group | T0 | T1 | -0.324 | 1.416 | -3.796 | 3.148 |
|  |  |  | T2 | 0.676 | 1.751 | -3.616 | 4.967 |
|  |  | T1 | T2 | 1.000 | 1.345 | -2.297 | 4.297 |
|  | Intervention group | T0 | T1 | -5.243^*^ | 1.416 | -8.715 | -1.771 |
|  |  |  | T2 | -1.892 | 1.751 | -6.183 | 2.400 |
|  |  | T1 | T2 | 3.351^*^ | 1.345 | 0.055 | 6.648 |
| Basic psychological needs | Control group | T0 | T1 | -0.216 | 1.024 | -2.727 | 2.295 |
|  |  |  | T2 | -0.081 | 1.158 | -2.919 | 2.757 |
|  |  | T1 | T2 | 0.135 | 0.960 | -2.219 | 2.489 |
|  | Intervention group | T0 | T1 | -2.541^*^ | 1.024 | -5.051 | -0.030 |
|  |  |  | T2 | -1.405 | 1.158 | -4.244 | 1.433 |
|  |  | T1 | T2 | 1.135 | 0.960 | -1.219 | 3.489 |

Note: * *P* < 0.05;

*MD*: Mean difference;

SE: Standard error;

CI: confidence interval

**Table S6.** Comparison of self-management behaviors scores in specific dimensions between the two groups at different time points.

| Indicators |  | T0 | T1 | T2 | | *F* | | *P* | | | *η^2^* | |
| --- | --- | --- | --- | --- | --- | --- | --- | --- | --- | --- | --- | --- |
| Medication management scores | Control group | 20.49 ± 3.11 | 21.65 ± 2.88 ^a^ | 20.97 ± 3.24 | | 3.407 | | 0.039 | | | 0.088 | |
|  | Intervention group | 21.49 ± 2.87 | 23.32 ± 2.15 ^a^ | 22.89 ± 2.16 ^a^ | | 6.910 | | 0.002 | | | 0.163 | |
|  | *F* | 2.06 | 8.051 | 9.007 | |  | |  | | |  | |
|  | *P* | 0.156 | 0.006 | 0.004 | |  | |  | | |  | |
|  | *η^2^* | 0.028 | 0.101 | 0.111 | |  | |  | | |  | |
|  | *F* _group effect_ = 8.596，*P* = 0.005；*F* _time effect_ = 9.517，*P* < 0.001；*F* _interaction effect_ = 0.801，*P* = 0.453 | | | | | | | | | | | |
| Dietary management | Control group | 33.22 ± 6.28 | 33.76 ± 6.13 | 32.14 ± 8.23 | | 1.375 | | 0.259 | | | 0.037 | |
|  | Intervention group | 32.86 ± 6.76 | 37.08 ± 5.89 ^a^ | 35.49 ± 5.63 ^a^ | | 12.301 | | < 0.001 | | | 0.257 | |
|  | *F* | 0.054 | 5.658 | 4.184 | |  | |  | | |  | |
|  | *P* | 0.818 | 0.020 | 0.044 | |  | |  | | |  | |
|  | *η^2^* | 0.001 | 0.073 | 0.055 | |  | |  | | |  | |
|  | *F* _group effect_ = 2.570，*P* = 0.113；*F* _time effect_ = 6.725，*P =* 0.002；*F* _interaction effect_ = 5.179，*P* = 0.007 | | | | | | | | | | | |
| Disease monitoring | Control group | 16.95 ± 3.23 | 17.62 ± 2.37 | 16.95 ± 2.81 | 2.126 | | 0.127 | | 0.057 | | | |
|  | Intervention group | 17.16 ± 2.06 | 19.00 ± 1.33 ^a^ | 18.41 ± 1.83 ^a^ | 8.639 | | < 0.001 | | 0.196 | | | |
|  | *F* | 0.118 | 9.489 | 7.009 |  | |  | |  | | | |
|  | *P* | 0.733 | 0.003 | 0.010 |  | |  | |  | | | |
|  | *η^2^* | 0.002 | 0.116 | 0.089 |  | |  | |  | | | |
|  | *F* _group effect_ = 5.396，*P* = 0.023；*F* _time effect_ = 9.718，*P* < 0.001；*F* _interaction effect_ = 2.977，*P* = 0.054 | | | | | | | | | | | |
| Emotion management | Control group | 23.81 ± 4.25 | 24.16 ± 3.96 | 24.22 ± 4.33 | 0.314 | | 0.732 | | | 0.009 | | |
|  | Intervention group | 24.84 ± 4.43 | 27.14 ± 3.21 ^a^ | 26.49 ± 3.10 ^a^ | 9.806 | | < 0.001 | | | 0.216 | | |
|  | *F* | 1.036 | 12.587 | 6.734 |  | |  | | |  | | |
|  | *P* | 0.312 | 0.001 | 0.011 |  | |  | | |  | | |
|  | *η^2^* | 0.014 | 0.149 | 0.086 |  | |  | | |  | | |
|  | *F* _group effect_ = 6.659，*P* = 0.012；*F* _time effect_ = 7.457，*P =* 0.001；*F* _interaction effect_ = 3.749，*P* = 0.026 | | | | | | | | | | | |
| Exercise management | Control group | 11.49 ± 2.28 | 11.32 ± 2.95 | 10.84 ± 2.34 | 1.770 | | 0.178 | | | 0.047 | | |
|  | Intervention group | 11.76 ± 2.76 | 13.32 ± 1.90 ^a^ | 12.43 ± 2.29 ^a^ | 8.565 | | < 0.001 | | | 0.194 | | |
|  | *F* | 1.036 | 12.587 | 6.734 |  | |  | | |  | | |
|  | *P* | 0.312 | 0.001 | 0.011 |  | |  | | |  | | |
|  | *η^2^* | 0.003 | 0.143 | 0.109 |  | |  | | |  | | |
|  | *F* _group effect_ = 7.148，*P* < 0.001；*F* _time effect_ = 4.721，*P =* 0.010；*F* _interaction effect_ = 5.981，*P* = 0.003. | | | | | | | | | | | |
| Daily life management | Control group | 13.86 ± 3.65 | 14.27 ± 3.26 | 14.30 ± 2.68 | 0.403 | | 0.670 | | | 0.011 | | |
|  | Intervention group | 14.19 ± 2.71 | 16.46 ± 3.13 ^a^ | 15.73 ± 2.82 ^a^ | 12.216 | | < 0.001 | | | 0.256 | | |
|  | *F* | 0.188 | 8.668 | 5.015 |  | |  | | |  | | |
|  | *P* | 0.666 | 0.004 | 0.028 |  | |  | | |  | | |
|  | *η^2^* | 0.003 | 0.107 | 0.065 |  | |  | | |  | | |
|  | *F* _group effect_ = 5.024，*P* = 0.028；*F* _time effect_ = 8.338，*P* = 0.001；*F* _interaction effect_ = 4.280，*P* = 0.018. | | | | | | | | | | | |
| Resource utilization | Control group | 16.92 ± 3.46 | 17.73 ± 3.63 | 17.89 ± 3.96 | 1.267 | | 0.288 | | | 0.034 | | |
|  | Intervention group | 18.03 ± 3.90 | 20.86 ± 3.75 ^a^ | 20.05 ± 3.01 ^a^ | 12.154 | | < 0.001 | | | | | 0.255 |
|  | *F* | 1.673 | 13.365 | 6.985 |  | |  | | | | |  |
|  | *P* | 0.2 | < 0.001 | 0.010 |  | |  | | | | |  |
|  | *η^2^* | 0.023 | 0.157 | 0.088 |  | |  | | | | |  |
|  | *F* _group effect_ = 9.080，*P* = 0.004；*F* _time effect_ = 10.000，*P* < 0.001；*F* _interaction effect_ = 3.421，*P* = 0.038 | | | | | | | | | | | |

Note: ^a^ a significant difference compared to T0 with Bonferroni correction (*P* < 0.017)

Sample size: 37 participants in both the intervention group and the control group

**Table S7.** Comparison of perceived social support scores in specific dimensions between the two groups at different time points.

| Indicators |  | T0 | T1 | T2 | *F* | *P* | *η^2^* |
| --- | --- | --- | --- | --- | --- | --- | --- |
| Friend support | Control group | 22.65 ± 4.50 | 22.78 ± 4.42 | 22.05 ± 4.74 | 0.796 | 0.455 | 0.022 |
|  | Intervention group | 24.03 ± 3.70 | 25.57 ± 2.88 ^a^ | 24.54 ± 3.43 | 4.809 | 0.011 | 0.119 |
|  | *F* | 2.072 | 10.308 | 6.679 |  |  |  |
|  | *P* | 0.154 | 0.002 | 0.012 |  |  |  |
|  | *η^2^* | 0.028 | 0.125 | 0.085 |  |  |  |
|  | *F* _group effect_ = 8.492，*P* = 0.005；*F* _time effect_ = 4.101，*P =* 0.021；*F* _interaction effect_ = 1.503，*P* = 0.229 | | | | | | |
| Family support | Control group | 20.43 ± 5.68 | 21.03 ± 5.03 | 20.92 ± 4.24 | 0.429 | 0.653 | 0.012 |
|  | Intervention group | 21.70 ± 4.45 | 23.43 ± 4.02 ^a^ | 22.92 ± 3.90 | 3.668 | 0.03 | 0.094 |
|  | *F* | 1.146 | 5.158 | 4.464 |  |  |  |
|  | *P* | 0.288 | 0.026 | 0.038 |  |  |  |
|  | *η^2^* | 0.016 | 0.067 | 0.058 |  |  |  |
|  | *F* _group effect_ = 4.078，*P* = 0.047；*F* _time effect_ = 3.290，*P =* 0.043；*F* _interaction effect_ = 0.043，*P* = 0.450 | | | | | | |
| Other support | Control group | 21.14 ± 3.64 | 20.73 ± 4.76 | 20.57 ± 4.64 | 0.38 | 0.685 | 0.022 |
|  | Intervention group | 22.57 ± 4.07 | 24.54 ± 3.56 ^a^ | 22.73 ± 3.98 ^b^ | 8.441 | 0.001 | 0.119 |
|  | *F* | 2.551 | 15.178 | 4.632 |  |  |  |
|  | *P* | 0.115 | < 0.001 | 0.035 |  |  |  |
|  | *η^2^* | 0.034 | 0.174 | 0.06 |  |  |  |
|  | *F* _group effect_ = 9.029，*P* = 0.004；*F* _time effect_ = 2.911，*P =* 0.058；*F* _interaction effect_ = 3.980，*P* = 0.021 | | | | | | |

Note: ^a^ a significant difference compared to T0 with Bonferroni correction (*P* < 0.017);

^b^ a significant difference compared to T1 with Bonferroni correction (*P* < 0.017)

Sample size: 37 participants in both the intervention group and the control group

**Table S8.** Comparison of basic psychological needs scores in specific dimensions between the two groups at different time points.

| Indicators |  | T0 | T1 | T2 | *F* | *P* | *η^2^* |
| --- | --- | --- | --- | --- | --- | --- | --- |
| Autonomy | Control group | 17.11 ± 2.71 | 17.19 ± 2.98 | 16.76 ± 3.15 | 0.465 | 0.630 | 0.013 |
|  | Intervention group | 17.92 ± 2.50 | 18.22 ± 2.23 | 17.49 ± 2.97 | 1.156 | 0.321 | 0.032 |
|  | *F* | 1.793 | 2.822 | 1.052 |  |  |  |
|  | *P* | 0.185 | 0.097 | 0.308 |  |  |  |
|  | *η^2^* | 0.024 | 0.038 | 0.014 |  |  |  |
|  | *F* _group effect_ = 2.523，*P* = 0.117；*F* _time effect_ = 1.872，*P =* 0.158；*F* _interaction effect_ = 0.126，*P* = 0.882 | | | | | | |
| Competence | Control group | 16.32 ± 3.05 | 16.32 ± 3.28 | 16.70 ± 3.14 | 0.64 | 0.530 | 0.018 |
|  | Intervention group | 17.19 ± 2.53 | 18.08 ± 2.78 | 18.14 ± 2.54 | 2.612 | 0.08 | 0.069 |
|  | *F* | 1.767 | 6.164 | 4.648 |  |  |  |
|  | *P* | 0.188 | 0.015 | 0.034 |  |  |  |
|  | *η^2^* | 0.024 | 0.079 | 0.061 |  |  |  |
|  | *F* _group effect_ = 5.307，*P* = 0.024；*F* _time effect_ = 2.055，*P =* 0.136；*F* _interaction effect_ = 1.197，*P* = 0.308 | | | | | | |
| Relatedness | Control group | 15.89 ± 3.19 | 16.03 ± 3.26 | 15.95 ± 3.24 | 0.04 | 0.961 | 0.001 |
|  | Intervention group | 16.84 ± 3.78 | 18.19 ± 2.42 ^a^ | 17.73 ± 2.82 | 3.269 | 0.044 | 0.084 |
|  | *F* | 1.354 | 10.472 | 6.391 |  |  |  |
|  | *P* | 0.249 | 0.002 | 0.014 |  |  |  |
|  | *η^2^* | 0.018 | 0.127 | 0.082 |  |  |  |
|  | *F* _group effect_ = 7.136，*P* = 0.009；*F* _time effect_ = 1.996，*P =* 0.143；*F* _interaction effect_ = 1.313，*P* = 0.275 | | | | | | |

Note: ^a^ a significant difference compared to T0 with Bonferroni correction (*P* < 0.017)

Sample size: 37 participants in both the intervention group and the control group
